# Electrical conductivity and vibrational studies induced phase transitions in [(C_2_H_5_)_4_N]FeCl_4_

**DOI:** 10.1039/c8ra07671e

**Published:** 2018-12-05

**Authors:** Kh. Ben Brahim, M. Ben gzaiel, A. Oueslati, M. Gargouri

**Affiliations:** Laboratory for Spectroscopic Characterization and Optics of Materials, Faculty of Sciences, University of Sfax B. P. 1171 3000 Sfax Tunisia oueslatiabderrazek@yahoo.fr

## Abstract

The compound, tetraethylammonium tetrachloroferrate [(C_2_H_5_)_4_N]FeCl_4_, was prepared by slow evaporation at room temperature. It was characterized by X-ray powder diffraction, thermal analysis, and impedance and vibrational spectroscopy. X-ray diffraction data confirmed formation of a single phase material which crystallized at room temperature in the hexagonal system (*P*6_3_*mc* space group). DSC showed the existence of two phase transitions at 413 K and 430 K. Electrical conductivity was measured in the temperature and frequency ranges of 390 K to 440 K and 40 Hz to 110 MHz, respectively. Nyquist plots revealed the existence of grains and grain boundaries that were fitted to an equivalent circuit. AC conductivity plots were analyzed by Jonscher's power law. Variations in the “*s*” values indicated that CBH models describe the conduction mechanism in regions I and II. Temperature dependence of Raman spectra showed that the most important changes were observed in the cationic parts ([(C_2_H_5_)_4_N]^+^). The activation energy value obtained from the line width decreased which indicated an order–disorder model.

## Abstract 2

Complex impedance spectra of [(C_2_H_5_)_4_N]FeCl_4_ at different temperature.

## Graphical abstract

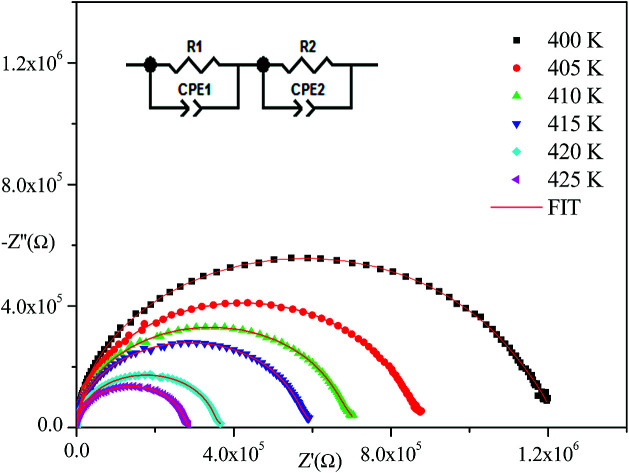

## 1. Introduction

Recently, material science has mainly concentrated on multifunctional materials. Obtaining such a material in the form of solids with a continuous lattice was found to be very complicated. However, a material with a multifunctional character can be obtained by building up hybrid solids formed by two lattices consisting of organic and inorganic parts. Much attention has been paid to organic–inorganic hybrid materials due to the possibility of each moiety contributing its own characteristic properties.^1–5^ This large family exhibits several phase transitions related to the reorientation dynamic of alkyl chains.^6–8^ In fact, they present interesting properties such as ferromagnetic, ferroelectric, ferroelastic, and thermal ones.^9–13^

Special attention is paid to compounds based on iron. Owing to the presence of the high-spin d^5^ in their molecules. Compounds containing tetrahalogenferrate(iii) units may also be used as attractive magnetic materials.^14,15^ Moreover, it was found that some of the iron(iii) complexes have been used in bioinorganic chemistry as substances for synthesizing model compounds, such as [Fe_2_S_2_Cl_4_]^2−^.^16^

With this aim, we wish to report the growth of [(C_2_H_5_)_4_N]FeC1_4_ crystals, and a description of basic information about their properties. This compound is of interest due to the presence of Fe(iii) in a tetrahedral coordination structure that mainly provides the existence of magnetic, electric, and elastic anomalies.^17^ At room temperature, the system belongs to the hexagonal system (space group *P*6_3_*mc*) with four formulae per unit cell, which has dimensions of *a* = *b* = 8.198 Å, *c* = 13.183 Å.^18^

In this contribution, the synthesis, X-ray powder diffraction, differential scanning calorimetry, impedance measurements, and vibrational study as a function of temperature were carried out.

## 2. Experimental details

### 2.1 Synthesis

Mixing a stoichiometric molar ratio of FeCl_3_ (purity 98%; FLUKA) and [N(C_2_H_5_)_4_]Cl (purity 97%; FLUKA) was performed in aqueous solution. After few days, yellow prismatic crystals were obtained by slow evaporation at room temperature. The sequence of the chemical reaction is the following:[(C_2_H_5_)_4_N]Cl + FeCl_3_ → [(C_2_H_5_)_4_N]FeCl_4_

### 2.2 Characterisations

Phase purity and homogeneity were checked using X-ray powder diffraction analysis. Using a Phillips PW 1710 powder diffractometer operating with CuK_α_ (*λ* = 1.5405 Å), the XRD pattern was recorded in a wide range of Bragg angles (10° ≤ 2*θ* ≤ 60°). Structural refinements ware carried out by the Rietveld refinement method.^19^ Differential scanning calorimetry, DSC, was executed with a Perkin Elmer DSC-7 instrument in a temperature range from 300 K to 470 K with a 5 K min^−1^ rate. Thermogravimetric analysis (TGA) was performed with an ATG-Q500 SETARAM in a heating process from room temperature up to 772 K with a heating rate of 10 K min^−1^.

To study the electrical proprieties, the [(C_2_H_5_)_4_N]FeCl_4_ powder was pressed at 5 t cm^−2^ ton pressure, forming a circular disc with a 1.1 mm thickness and 8 mm diameter. Measurements were executed in the frequency range of 40 Hz to 110 MHz using a 4294A impedance analyzer. The measurements were carried out under an excitation voltage of 50 mV. In the present study, we found that after several tests our pellet became softer after 430 K, which proved that these organic–inorganic crystals of tetraethylammonium tetrachloroferrate show plastic behavior at high temperature.^6^ Therefore, the sample is not in the form of an electrical dipole, which shows that measurement after 430 K is not possible.

Using a Perkin Elmer FT-IR 1000 spectrophotometer, the infrared spectrum of [(C_2_H_5_)_4_N]FeCl_4_ was recorded at room temperature in the frequency range of 500–3500 cm^−1^.

Raman scattering measurements were analyzed by a Horibe Jobin-Yvon T64000 spectrometer over the frequency range 50–3500 cm^−1^ with a resolution of 3 cm^−1^ in the temperature range 303–428 K. The program LABSPEC5 software was used for fitting the spectra with a combination of Lorentzian–Gaussian functions. The fitting procedure was performed in order to quantitatively analyze the evolution of Raman bands as a function of temperature.

## 3. Results and discussion

### 3.1 X-ray powder diffraction analysis

**Fig. 1:**
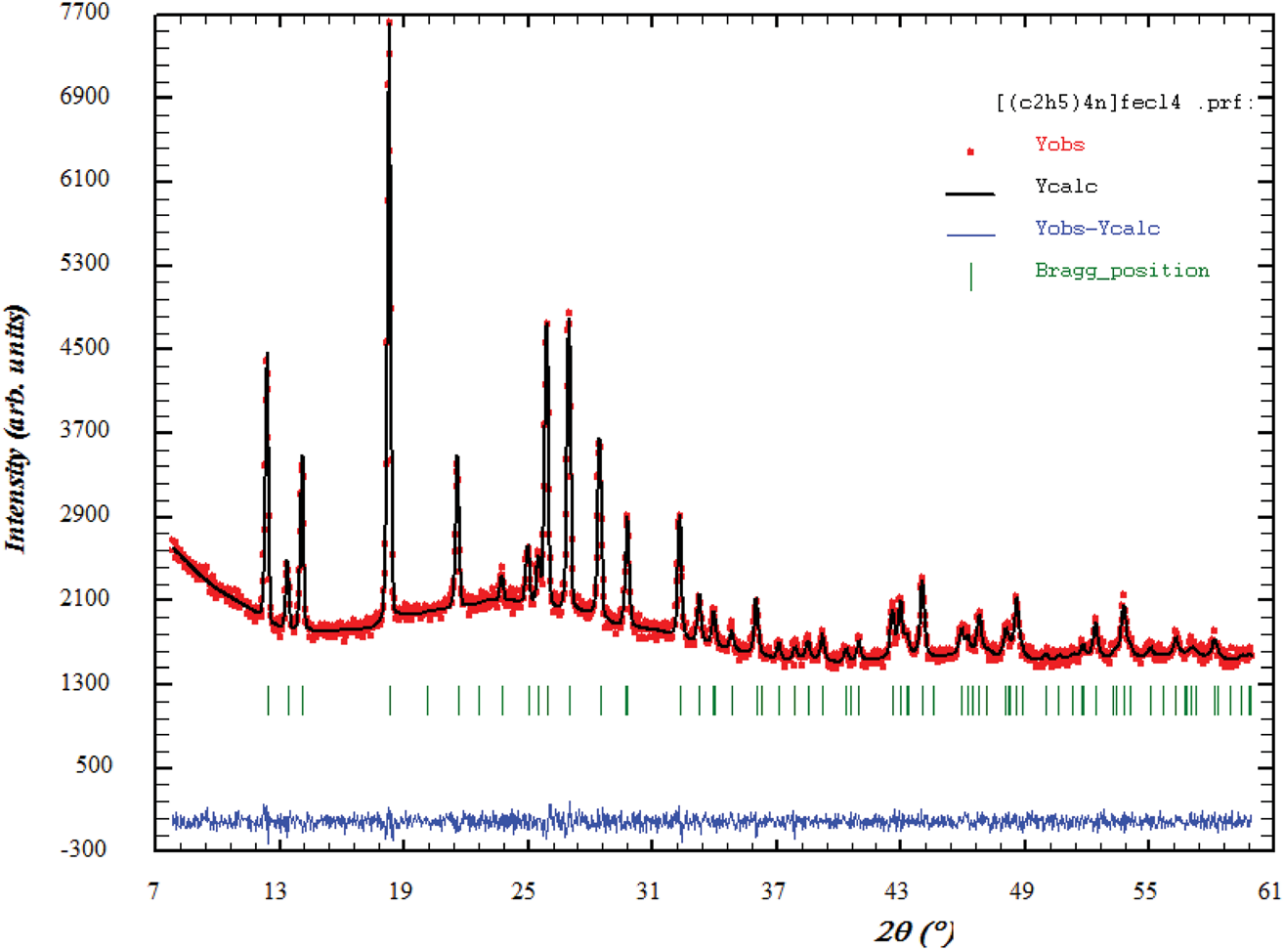
X-ray diffractogram of [(C_2_H_5_)_4_N]FeCl_4_ in the 2*θ* range 10–60°. The red circle indicates the experimental data and calculated data are represented by black continuous lines. The lowest curve in blue shows the difference between experimental and calculated patterns. The green vertical bars describe the Bragg position.

Fig. 1 shows the XRD patterns of [(C_2_H_5_)_4_N]FeCl_4_. The Rietveld refinement was performed using the FULLPROOF software program. The circles symbolize the experimental data and the line presents the simulated pattern. The purity of the studied compound was proved and the reflection peaks were indexed in the hexagonal system with the *P*6_3_*mc* space group. The corresponding lattice parameters were found to be: *a* = *b* = 8.218 Å, *c* = 13.203 Å and the cell volume was *V* = 772.2 Å^3^. The quality of the refinement was evaluated through the goodness of fit where *χ*^2^ = 1.23. The values of the reliability factors obtained from the refinement are *R*_ωp_ = 13.5, *R*_p_ = 18.5 and *R*_exp_ = 12.04 and the fitted parameters were found to be in a good agreement with those in the literature.^18^

### 3.2 Thermal analysis

**Fig. 2:**
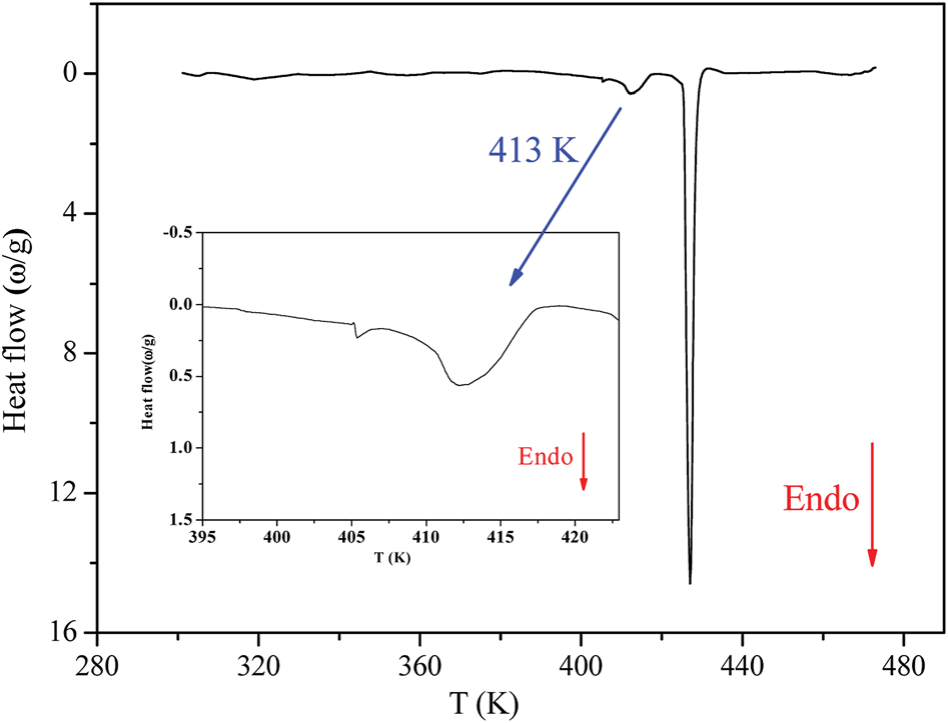
Differential scattering calorimetric trace of [(C_2_H_5_)_4_N]FeCl_4_.

Fig. 2 shows the DSC thermogram for [(C_2_H_5_)_4_N]FeCl_4_. The DSC curve was recorded with a heating scan at 5 K min^−1^. The calorimetric measurement showed that the compound under investigation undergoes discontinuation of two phase transitions at *T*_1_ = 413 K and *T*_2_ = 430 K. The characteristic dynamical values of these phase transitions are listed in Table 1.

**Table 1:** The characteristic dynamical values of [N(C_2_H_5_)_4_]FeCl_4_

*T* (K)	Δ*H* J mol^−1^	Δ*S* J mol^−1^ K^−1^
413	459	1.112
430	545	1.267

From the Boltzmann equation, (Δ*S* = *R* ln(*Ω*)), where *Ω* is the rapport number of distinguishable orientations allowed in the high- and the low temperature phases (*N*_1_/*N*_2_).

The obtained values of *Ω* were 1.14 and 1.16 at *T*_1_ and *T*_2_, respectively (*Ω*_1,2_ < 2), which reveals that these two phase transitions are not purely “order–disorder”.^20^

**Fig. 3:**
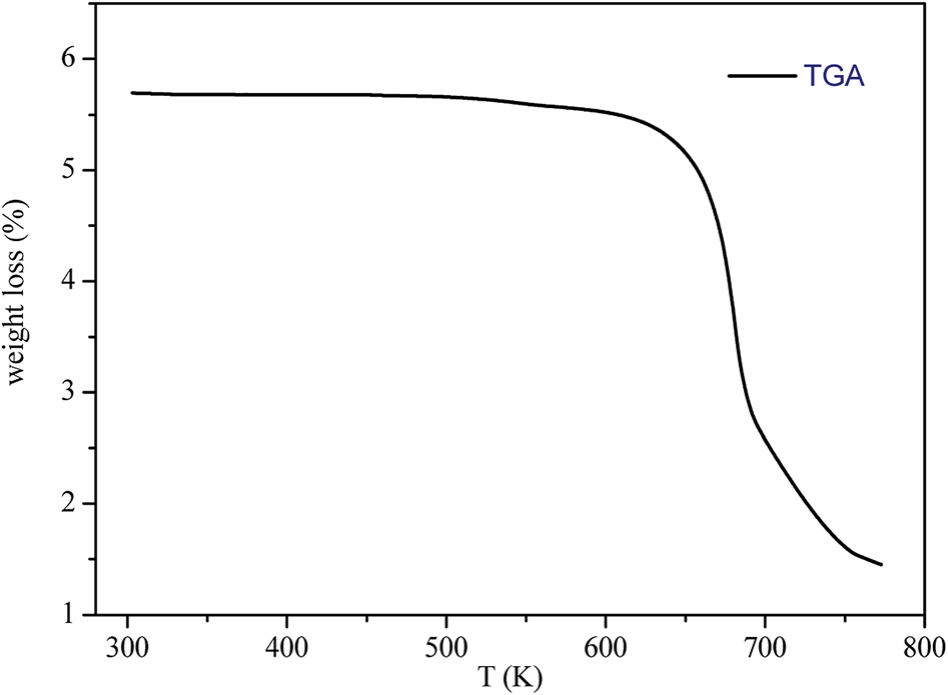
TGA curve of [(C_2_H_5_)_4_N]FeCl_4_.

The thermogravimetric analysis (TGA) curve in Fig. 3 shows that this compound started to decompose at 615 K. Besides, no weight losses were observed between 300 and 615 K.

### 3.3 Electrical measurements

Impedance spectroscopy is one of the most suitable techniques to investigate electrical conductivity and to analyze the charge transport processes in grains, grain boundaries, and electrode effects in materials in terms of thermal and frequency ranges.^21^

**Fig. 4:**
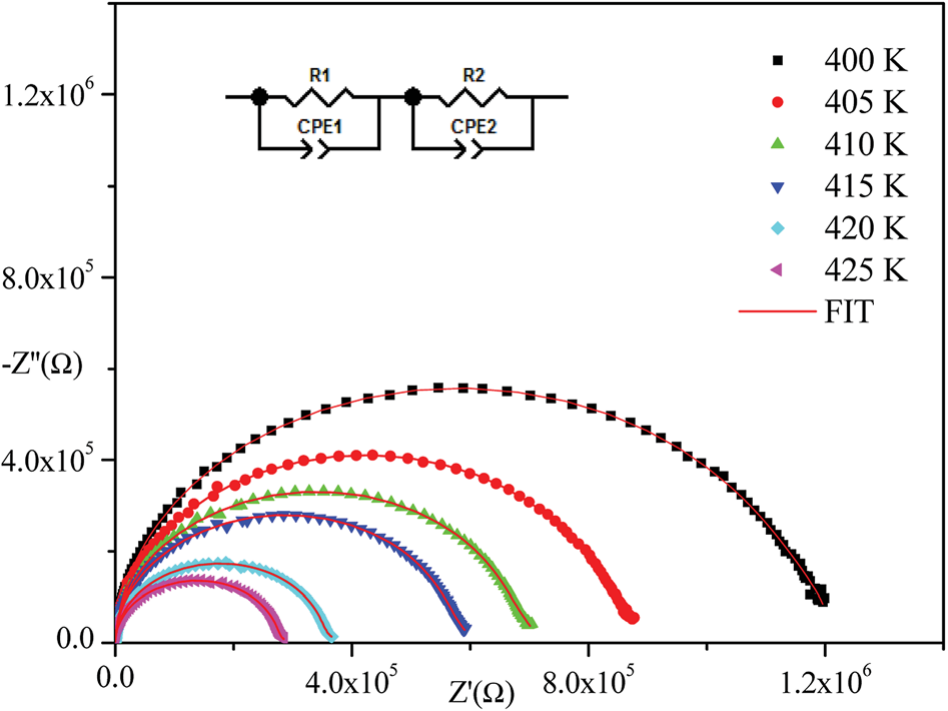
Complex impedance spectra of [(C_2_H_5_)_4_N]FeCl_4_ at different temperatures.

Fig. 4 displays the obtained Nyquist plots from experimental results at several temperatures. With respect to these curves, the experimental points show semi-circles where their diameters decrease with temperature increase indicating that the conduction mechanism is thermally activated.^22^ In addition, they are centered below the abscissa axis indicting that the relaxation process in the material is of a non-Debye type.^23^ (The Debye model presents the ideal response^24^ where all the dipoles participating in the relaxation phenomenon have the same relaxation time *τ*). The Nyquist diagram is a perfect semicircle centered on the abscissa axis but there are other cases where all dipoles do not have the same relaxation time (distribution of relaxation time). There are several empirical models derived from the Debye equation that correctly describe these types of relaxations including the Cole–Cole model^25–27^ and the Nyquist plot described by a flattened arc with its center located below the real axis on a line deviated from the angle of (1 − *α*)π/2 where *α* describes the interactions between dipoles.

Nyquist plots were analyzed using Zview Software. The best fits were obtained using an equivalent circuit (inset Fig. 4) realized by two cells which consisted of a parallel combination of bulk resistance *R*_1_ and fractal capacity CPE_1_ in series with a parallel combination of grain boundary resistance *R*_2_ and fractal capacity CPE_2_. The constant phase element (CPE) impedance is given by the following equation:^28^1
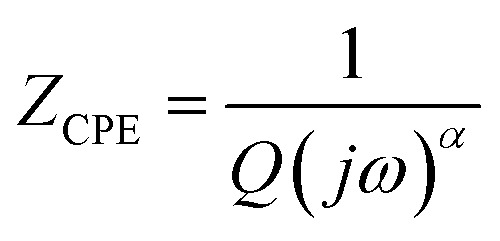
where *Q* presents the capacitance value of the CPE fractal capacity and *α* relates to the deviation degree with respect to the pure capacitor value.

The theoretical impedance of real (*Z*′) and imaginary (−*Z*′′) parts linked to the equivalent circuit were calculated according to the following eqn (2) and (3):2
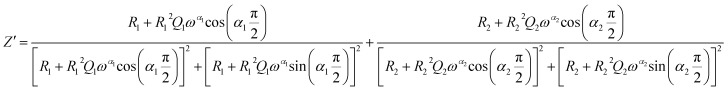
3
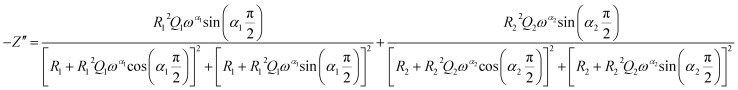


**Fig. 5:**
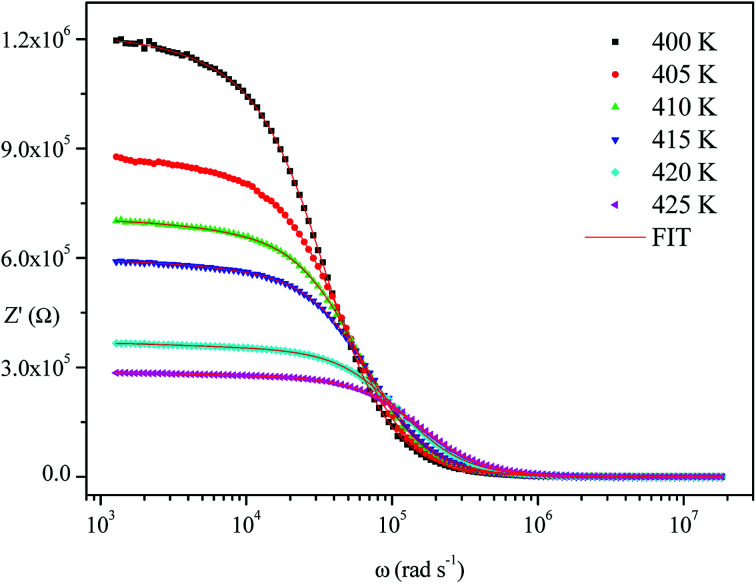
Variation of the real part of impedance as a function of frequency and temperature.

**Fig. 6:**
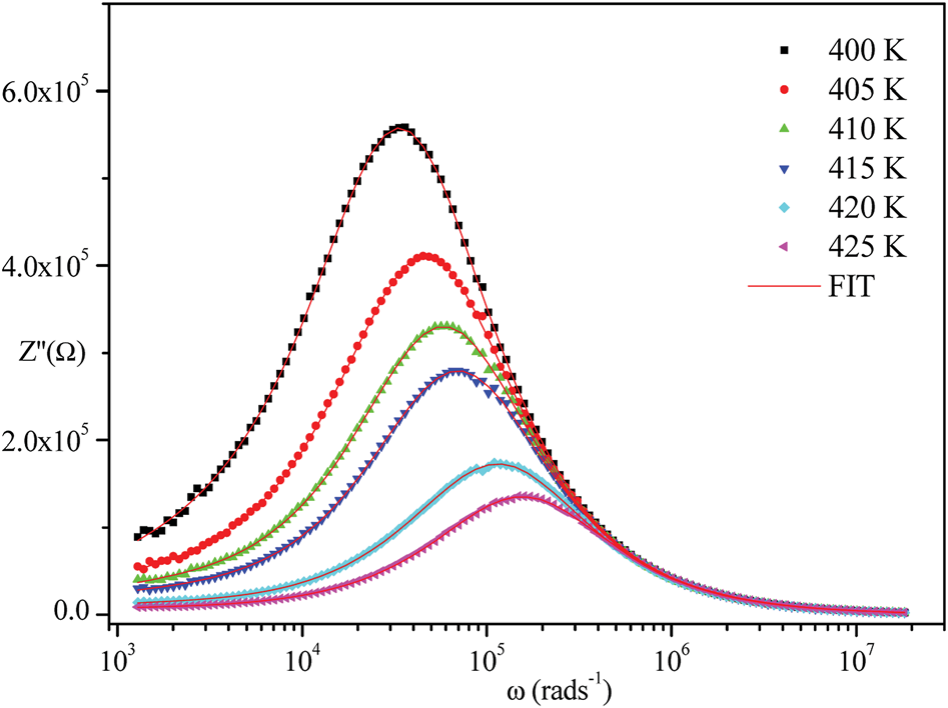
Variation of the imaginary part of impedance as a function of frequency at various temperatures.

The frequency dependence of the impedance of real (*Z*′) and imaginary (−*Z*′′) at various temperatures, are depicted in Fig. 5 and 6, respectively. It was noted that the magnitude of *Z*′ decreased, where increases in both frequency and temperature indicated an increase in AC conductivity of the sample. At frequencies higher than 10^6^ Hz, the coincidence of the impedance (*Z*′) at all temperatures showed a possible freeing of space charge^29^ whereas, the *Z*′′ peak maximum decreased with a rise in temperature. The peak frequency *ω*_max_ shifted to higher values and the later reflected a non-Debye type relaxation.^30^

Fig. 4–6 show good conformity between experimental data and the theoretical line indicating that the equivalent circuit described the electric properties of the investigated compound quite well. As a matter of fact, details of fitted values for different temperatures are gathered in Table 2. It is obvious that all the capacitance values (*Q*_1_ and *Q*_2_) are in the range of picofarads (pF) and nanofarads (nF), respectively, implying a single semicircle response is from grain interiors and grain boundaries. The values of *α* vary in the range of 0.81–0.99 showing a capacitive behavior of the fractal phase CPE.^31^

**Table 2:** Fitted circuit parameters at different temperature

*T* (K)	*R*_1_ (×10^5^)	*Q*_1_ (×10^−11^)	*α*_1_	*R*_2_ (×10^4^)	*Q*_2_ (×10^−9^)	*α*_2_
400	10.86	2.856	0.989	12.786	1.943	0.958
405	8.159	2.716	0.993	7.169	6.244	0.916
410	6.597	2.677	0.994	5.358	9.588	0.900
415	5.611	2.654	0.994	4.119	16.94	0.882
420	3.484	2.572	0.996	2.349	41.18	0.833
425	2.726	2.406	0.971	1.562	61.70	0.810

**Fig. 7:**
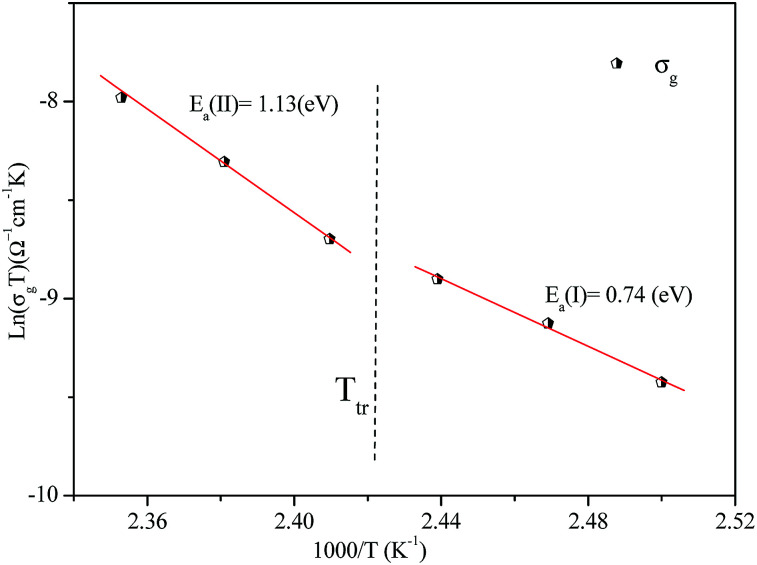
Plot of ln(*σ*_g_*T*) *versus* 1000/*T* for the [(C_2_H_5_)_4_N]FeCl_4_ crystal.

Electrical conductivity spectroscopy is a well-founded method for characterizing hopping dynamics of the charge carriers. Based on the equivalent circuit parameters, grain conductivity is expressed according to the following expression:^29^4
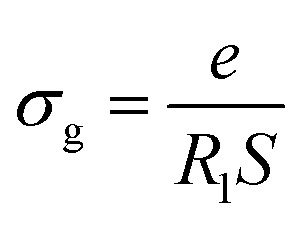
where *e* and *S* are the thickness and area of the sample, respectively, and *R*_1_ is the bulk resistance. The dependence of ln(*σ*_g_*T*) *versus* 1000/*T* is presented in Fig. 7. The experimental data evinced a linear variation whose *σ*_g_ decreased as temperature decreased. This indicated that electrical conduction in the material is a thermally activated process and was fitted well by the Arrhenius relation as described by eqn (5):5
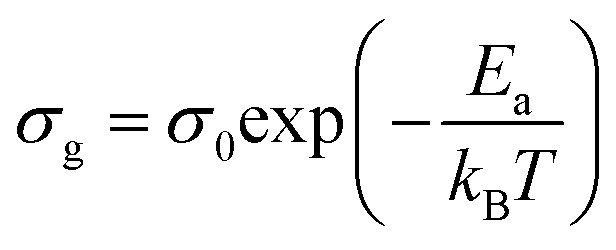
where *σ*_0_ is the pre-exponential factor, *E*_a_ is the activation energy, and *k*_B_ is the Boltzmann constant.

The activation energies obtained by the linear fit at *T*_1_ (413 ± 5 K) are *E*_a_ (I) = 0.74 eV in region I and *E*_a_ (II) = 1.13 eV in region II. The observed variation in the activation energies between the two temperature domains can be explained by movement of the cationic and anionic parts with temperature.^32^ However, the discontinuity at 413 ± 5 K agrees with the phase transition observed in the DSC measurements.

### 3.4 AC conductivity studies

**Fig. 8:**
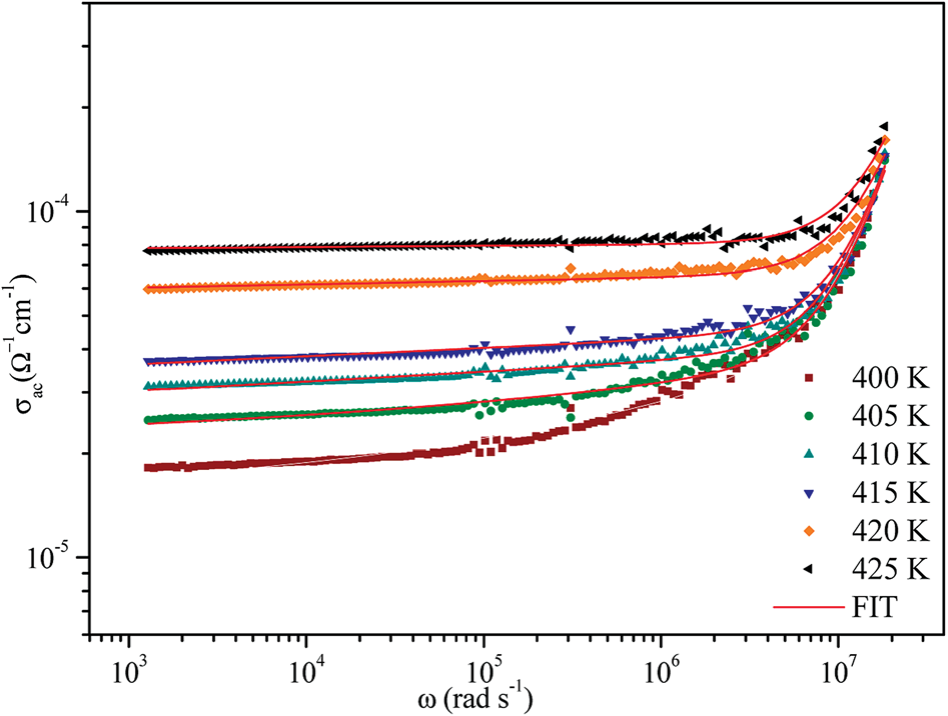
Variation of *σ*_ac_*versus* 1000/*T* at different frequencies.

The variation of AC conductivity with angular frequency for [(C_2_H_5_)_4_N]FeCl_4_ is shown in Fig. 8 at different temperatures. Each conductivity plot exhibits two distinct regions. First, it remained almost constant at lower frequencies; this plateau corresponds to dc conductivity. It is seen that an increase of temperature is closely related to an increase of the AC conductivity which suggests that electrical conduction in the material is a thermally activated process and suggests semiconductor properties of this compound. Second, at higher frequencies, the AC conductivity dependence occurred with changes in slope and increased gradually with an increase in frequency.

Besides, the dispersive region shifted to higher frequencies with an increase in temperature. This phenomenon of conductivity dispersion is generally analyzed using Jonscher's universal power law^33^ expressed by eqn (6):

6 *σ*_ac_(*ω*) = *σ*_dc_ + *Aω*^*s*^

where *σ*_dc_ is dc conductivity, *A* is a frequency independent pre-exponential factor that depends only on temperature and *s* is the dimensionless frequency exponent having a value between 0 and 1; *s* is the degree of interaction between mobile ions with the environments surrounding them.

**Fig. 9:**
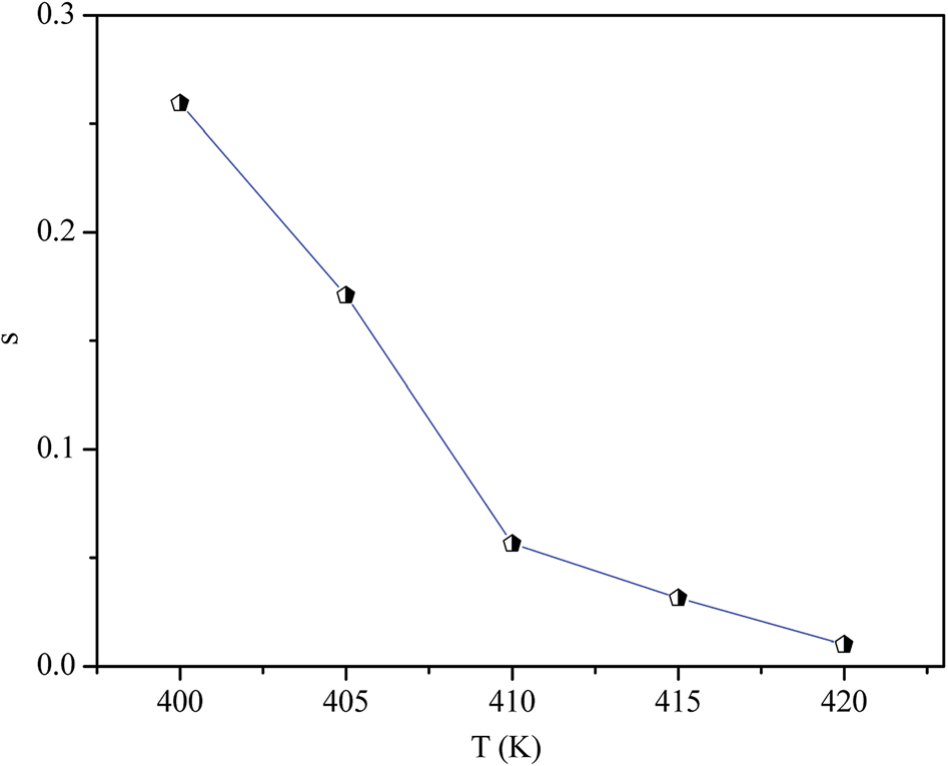
Temperature dependence of the frequency exponent *s*.

In order to identify the predominant conduction mechanism for [(C_2_H_5_)_4_N]FeCl_4_, different theoretical models proposed by Long *et al.* were used to correlate the conduction mechanism of AC conductivity with the predicted exponent *s*(*T*) behavior.^34–37^ These different models are: QMT, the quantum mechanical tunneling model, OLPT, the overlapping large polaron tunneling model, NSPT, the non-overlapping small polaron tunneling model, and CBH, the correlated barrier-hopping model. Comparing the obtained results with the extracted exponent *s* temperature dependence behavior (Fig. 9) for the different models, the conduction mechanism for [(C_2_H_5_)_4_N]FeCl_4_ was best interpreted with the correlated barrier hopping (CBH) model in region I and II.

According to this model of conduction^38^ (CBH), the thermal dependence of S, is given by eqn (7):7
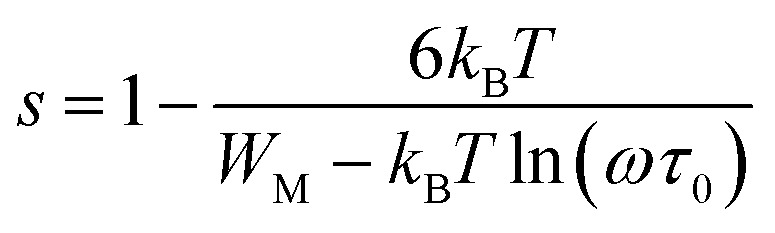
where *W*_M_ is the polaron binding energy and *τ*_0_ is the characteristic relaxation time. By a first approximation (at higher binding energy), eqn (8) is written in the form:8
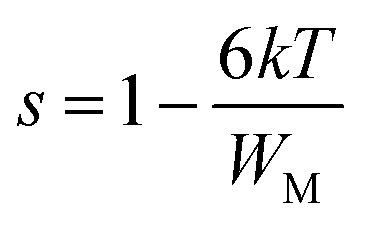


The AC conductivity as a function of frequency (*σ*_ac_(*ω*)) can be expressed according to this model^39,40^ by eqn (9):9
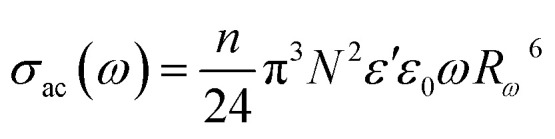
where *n* is the number of polarons participating in the hopping process (*n* = 1 for single polarons or 2 for bipolarons), *N* is the density of localized states in which carriers exist and *R*_*ω*_ is the hopping distance for the condition *ωτ* = 1 which is calculated using eqn (10):10
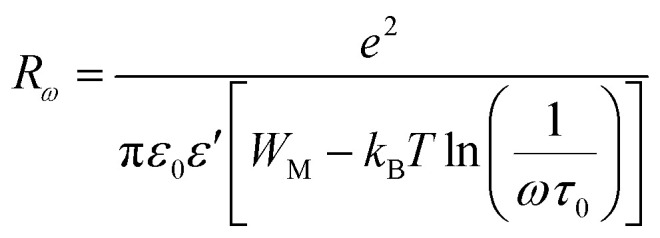


**Fig. 10:**
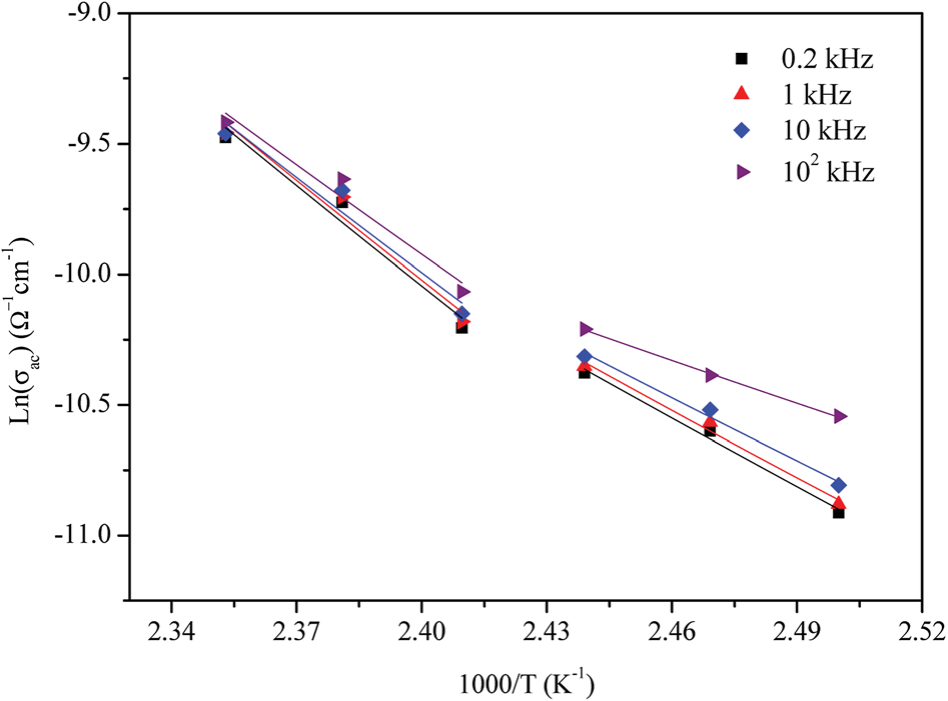
Temperature dependence of ln(*σ*_ac_) at different frequencies.

The potential barrier *W*_M_ was calculated from the linear fit of the curve (1s) as a function of temperature from eqn (8). In the general case, if *W*_M_ = *E*_a_/2, then bipolaron hopping is the dominating conduction mechanism and if *W*_M_ = *E*_a_/4, then a single polaron is dominating. The obtained values of *W*_M_ are 0.12 eV in region I and 0.25 eV in region II. These values are proximate to *E*_a_/4, which proves that single polaron hopping is the dominating conduction mechanism in the material. Temperature dependence of conductivity (ln(*σ*_ac_)) at different frequencies is shown in Fig. 10. There is good agreement between experimental and theoretical curves. The density of localized states (*N*) obtained by fit are understood between 5.45 × 10^34^ to 5.12 × 10^32^ cm^−1^ in region I and between 3.02 × 10^32^ to 3.7 × 10^31^ cm^−1^ in region II. From a qualitative analysis of these values, we noted that the density of localized states decreased with increasing frequency. This reduction is explained by an increase in the disorder which decreased stabilities of states with non-localization of the latter.^41^

### 3.5 Vibrational study at room temperature

**Fig. 11:**
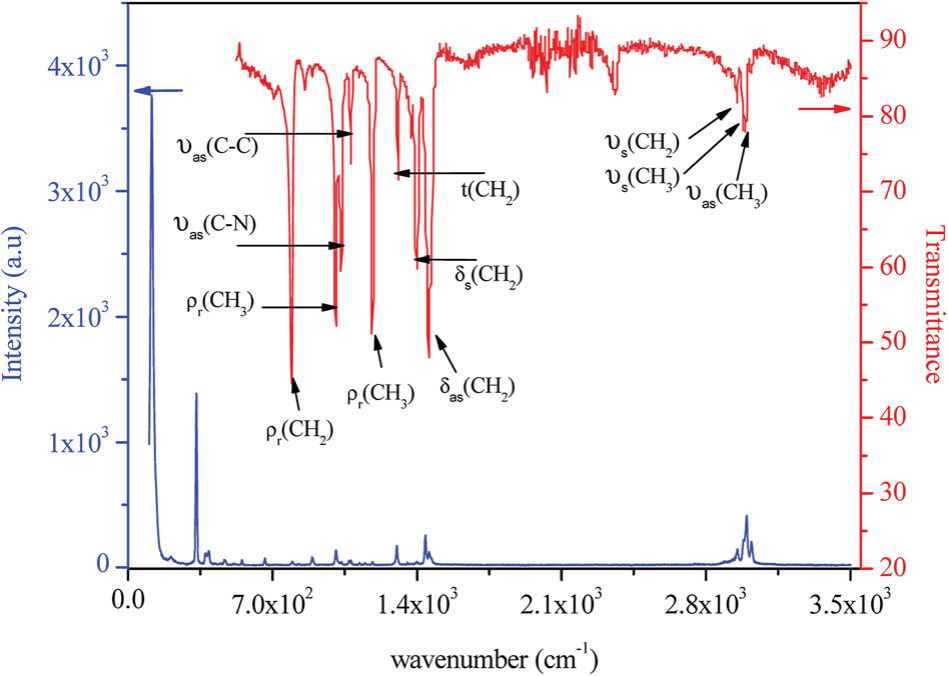
Experimental IR and Raman spectra of [(C_2_H_5_)_4_N]FeCl_4_ at room temperature.

The IR and Raman spectra of tetraethylammonium tetrachloroferrate are presented in Fig. 11. Tentative assignments for a majority of the vibrational bands using previously reported data in the literature on similar compounds are: [(C_2_H_5_)_4_N]InBr_4_,^42^ [(C_2_H_5_)_4_N]SbF_6_,^43^ [(C_2_H_5_)_4_N]SbCl_6_,^44^ [(C_2_H_5_)_4_N]_2_CoCl_4_,^45^ [(C_2_H_5_)_4_N]_2_HgI_4_, [(C_2_H_5_)_4_N]Hg_3_I_8_,^46^ and [(C_6_H_5_)_4_P]FeCl_4_ (ref. 47) and are listed in Table 3.

**Table 3:** Observed Raman and IR frequencies in (cm^−1^) of [N(C_2_H_5_)_4_]FeCl_4_^a^

[(C_2_H_5_)_4_N]FeCl_4_		
Raman (cm^−1^)	IR (cm^−1^)	Assignment
3022	—	*ν*_as_(CH_3_)
2990	2989	*ν*_as_(CH_2_)
—	2979	*ν*_s_(CH_3_)
2952	2950	*ν*_s_(CH_2_)
2937	—	
2890	—	
1460	1455	*δ*_as_(CH_2_)
1441	—	
1400	1398	*δ*_s_(CH_2_)
1353	—	*δ*_s_(CH_3_)
1302	1307	t(CH_2_)
1185	1183	ρ_r_(CH_3_)
1150	—	
1124	—	
1082	1079	*ν*_as_(C–C)
1071	—	
1052	—	
1022	1033	*ν*_as_(C–N)
1008	1006	ρ_r_(CH_3_)
895	—	*ν*_s_(C–C)
794	791	ρ_r_(CH_2_)
665	—	*ν*_s_(N–C)
554	—	*δ*_s_(CNC)
516	—	
397	—	*ν*_3_(FeCl_4_)
379	—	*ν*_3_(FeCl_4_)
335	—	*ν*_1_(FeCl_4_)
142	—	*ν*_4_(FeCl_4_)
121	—	*ν*_2_(FeCl_4_)

a *ν*_s_, *ν*_1_: Symmetric stretching; *ν*_as_, *ν*_3_: asymmetric stretching; *δ*_s_, *ν*_2_: symmetric bending; *δ*_as,_*ν*_4_: asymmetric bending; *t*: twitching; *ρ*_r_: rocking.

#### Vibrations of the [FeCl_4_]^−^ anions

Symmetric and asymmetric bending vibrations (*ν*_2_(FeCl_4_) and *ν*_4_(FeCl_4_)) are observed at 121 and 142 cm^−1^, respectively. The strongest Raman band at 335 cm^−1^ is assigned to the symmetric stretching modes (*ν*_1_(FeCl_4_)), whereas the asymmetric stretching vibration (*ν*_3_(FeCl_4_)) gives the Raman peaks at 379 and 397 cm^−1^.

#### The internal vibrations of the [(C_2_H_5_)_4_N]^+^ cations

The Raman spectrum above 3000 cm^−1^ displays one band (at *ca.* 3022 cm^−1^) attributable to the (*ν*_as_ (CH_3_)) vibration. The Raman and IR bands at 2990 and 2989 cm^−1^, respectively, are attributed to the CH_2_ asymmetric stretching mode (*ν*_as_(CH_2_)). The IR peak at 2979 cm^−1^ is assigned to the CH_3_ symmetric bending mode (*δ*_s_(CH_3_)). On the other hand, the symmetric bending mode (*δ*_s_(CH_2_)) is observed at 2952 and 2950 cm^−1^ in the Raman and IR spectra, respectively. The asymmetric bending of CH_2_ groups (*δ*_as_(CH_2_)) is found at 1460 and 1440 cm^−1^ in the Raman and at 1455 cm^−1^ in the IR spectra. The CH_3_ symmetric bending (*δ*_s_(CH_3_)) mode appears at 1353 cm^−1^ in the Raman spectrum. The observed Raman band at 1307 cm^−1^ and IR band at 1302 cm^−1^ are assigned to CH_2_ twitching mode (*t*(CH_2_)). The CH_3_ rocking vibration (*ρ*_r_(CH_3_)) is in the Raman spectrum at 1185 and 1008 cm^−1^ and also as doublet IR bands at 1183 and 1006 cm^−1^. The asymmetric stretching mode (*ν*_as_(C–C)) is identified at 1082 cm^−1^ in Raman and at 1079 in IR, whereas the symmetric stretching mode (*ν*_s_(C–C)) presents a Raman band at 895 cm^−1^. The N–C asymmetric stretching vibration (*ν*_as_(N–C)) is observed at 1022 and at 1033 cm^−1^ in Raman and IR, respectively, while the symmetric bending modes (*ν*_s_(N–C)) are located at 665 cm^−1^ in the Raman spectrum. A strong transmission band appearing in IR near 791 cm^−1^ and Raman band at 794 cm^−1^ are assigned to the CH_2_ rocking vibration (*ρ*_r_(CH_2_)). The band related to the CNC skeleton deformation (*δ*_s_(CNC)) is detected in the Raman spectrum at 554 cm^−1^.

### 3.6 Temperature dependence of the Raman spectra

In order to gain more information on the crystal dynamics and on the mechanism involved in the transition, we undertook a vibrational study using Raman scattering between 303 and 428 K.

**Fig. 12:**
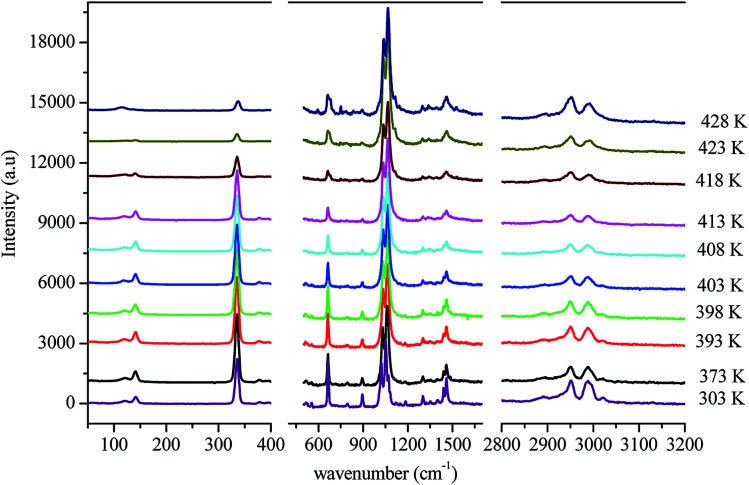
Evolution of the Raman spectrum as a function of temperature.

Fig. 12 illustrates the Raman spectra of [(C_2_H_5_)_4_N]FeCl_4_ recorded in three spectral ranges ([50–400 cm^−1^], [500–1600 cm^−1^], and [2800–3200 cm^−1^]). A deconvolution of all spectra was necessary to follow the temperature dependence of the wavenumber and half maximum. This deconvolution was carried out by means of Labspec software with a combination of two Lorentzian and Gaussian functions.

Fig. 14(a and b) shows the temperature dependence of the position and half maximum for some bands issued from the internal modes of the anionic part [FeCl_4_]^−^ observed between 50 and 400 cm^−1^. We noticed that this figure indicates weak changes near 418 K where the phase transition has been observed. The line at 121 cm^−1^ associated with *ν*_2_(FeCl_4_) mode underwent a shift toward high frequency by 2 cm^−1^, with a half maximum decrease by 5 cm^−1^. The two bands at 379 and 397 cm^−1^ attributed to *ν*_3_(FeCl_4_) vanished at the temperature transition.

**Fig. 13:**
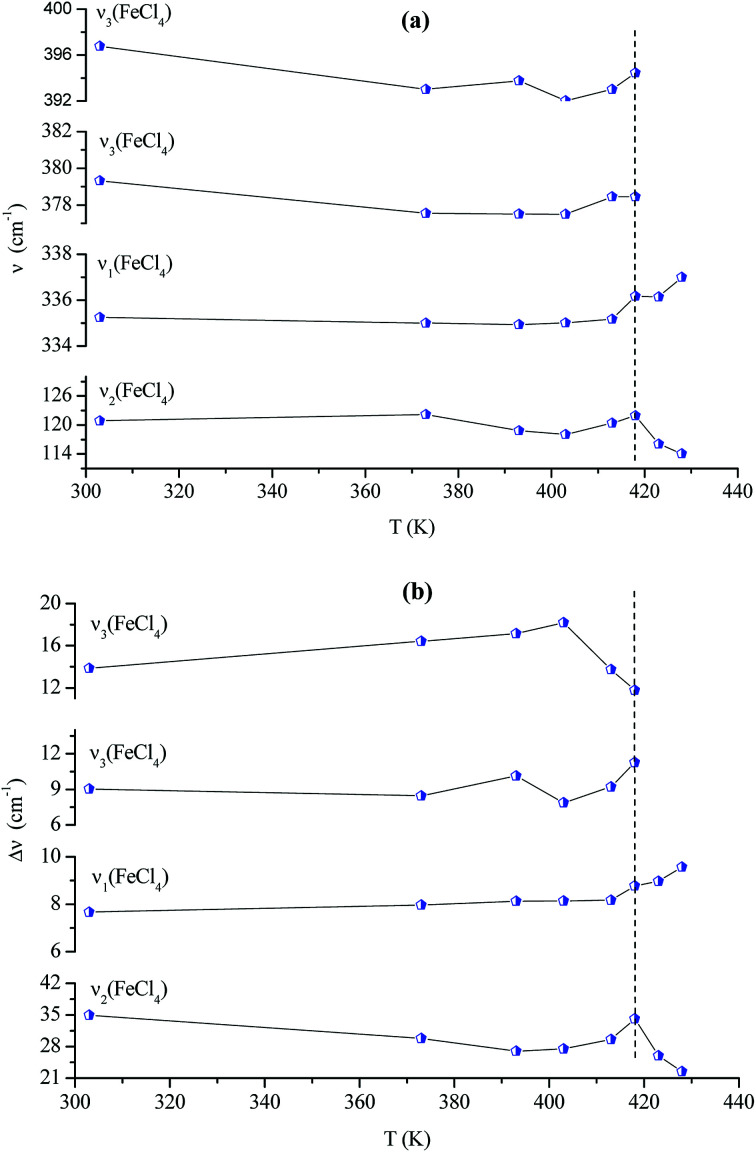
Temperature dependence of some Raman position (a) and half-maximum (b) associated with the inorganic part.

In Fig. 13(a and b), significant changes were obtained for some peaks located in the 500–1600 cm^−1^ frequency range which issued from the internal modes of the cationic parts. The two bands around 554 and 1185 cm^−1^ assigned to the *δ*_s_(CNC) and *ρ*_r_(CH_3_), respectively, vanished after the phase transition (418 K), while three bands appeared in the vicinity of 595, 750, and 1528 cm^−1^ at 418 K. A large change was detected for the peak at 665 cm^−1^ (*ν*_s_(N–C)), where it broke into two bands after 418 K. Another important change in the position was observed near 1440 and 1460 cm^−1^ (*δ*_as_(CH_2_)). These two bands merged into a single line at 418 K while the half maximum of them increased to 15 cm^−1^ above the transition. The bands observed between 2800 and 3200 cm^−1^, which were assigned to the symmetric and asymmetric vibration of CH_2_ and CH_3_ groups, did not change significantly with temperature; their position and half maximum underwent weak changes (a few cm^−1^) which means that these modes are not directly connected to a phase transition.

**Fig. 14:**
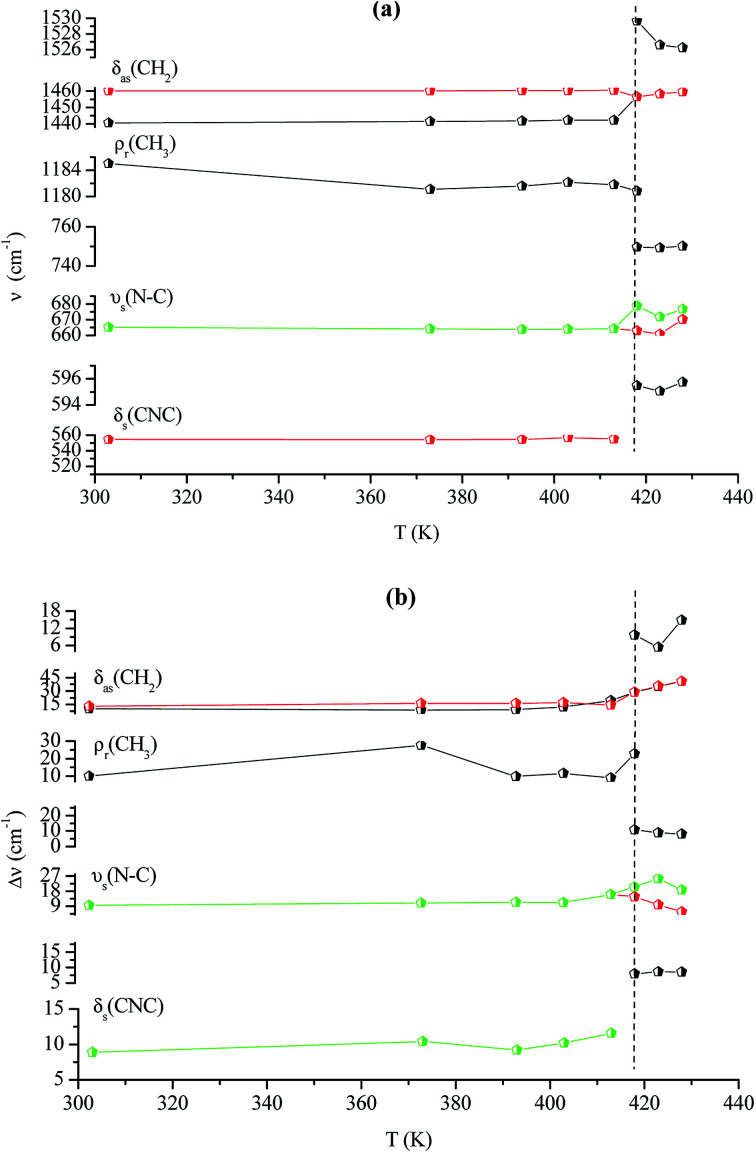
Temperature dependence of some Raman position (a) and half-maximum (b) associated with the organic part.

Spectral anomalies observed in Raman spectra of the [(C_2_H_5_)_4_N]FeCl_4_ crystals are due to a phase transition at *T* = 418 K which is in good agreement with DSC measurements and electrical proprieties. The most pronounced changes were found to be connected with vibration of the cationic part. In order to confirm the main role of the organic part in the phase transition point, we focused our study on the band around 1440 cm^−1^ associated with the CH_2_ asymmetric bending mode *δ*_as_(CH_2_).

#### 3.6.1 Temperature dependence of the Raman wavenumbers

According to Andrade and Porto,^48^ the thermal dependence of the Raman shift of a phonon linked to an order–disorder mechanism can be expressed by eqn (11):

11 *ν*^2^ = *ν*_0_^2^[1 + *γ*(*T* − *T*_t_)]

where *ν*_0_ is the so-called ‘hard-core wavenumber’ at *T* = *T*_tr_, *γ* is a thermal coefficient of the substance, and *T*_tr_ is the transition temperature. In the general case where *γ* is very weak, then eqn (11) can be written^49^ as eqn (12):12
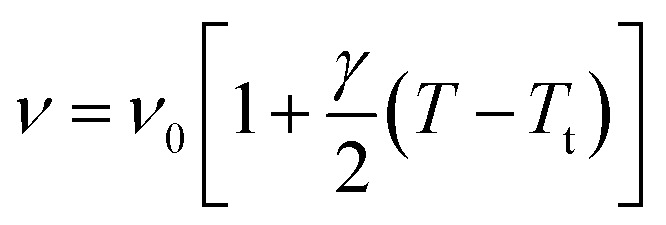


The thermal coefficient *γ* associated with variation of the crystal volume and the wavenumber is given by eqn (13):13
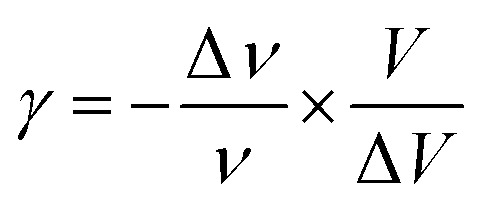
where *ν* is the band position and *V* is the volume of the crystal at room temperature.

**Fig. 15:**
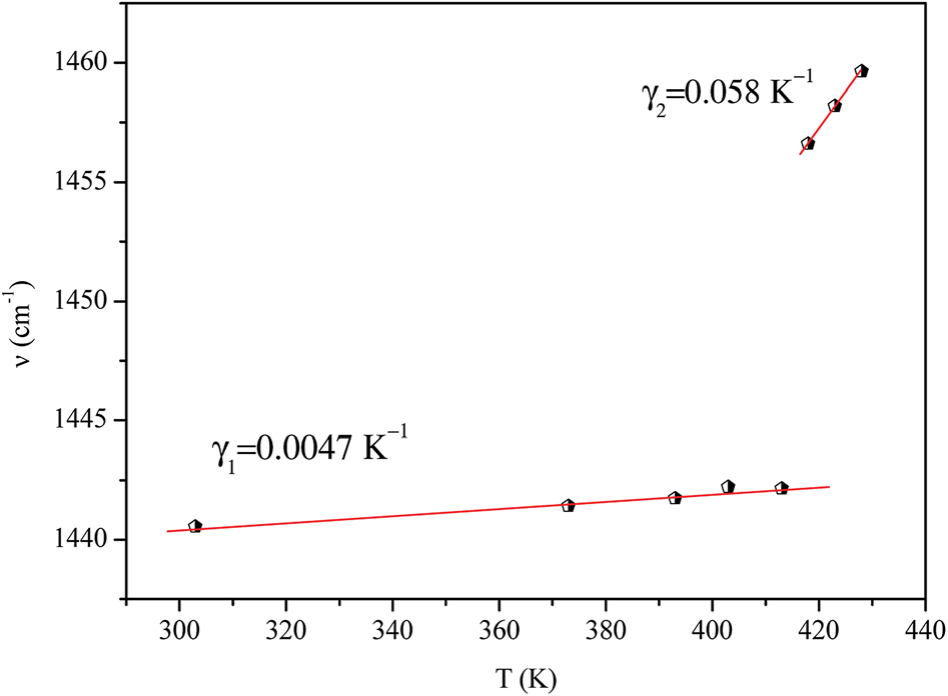
Temperature dependence of the band position at 1440 cm^−1^.

We adjusted the evolution of the frequency of the studied modes using eqn (12) in the temperature range that extends above and below the phase transition temperature (Fig. 15). The variation of the frequency shows two linear regimes, one below and the other above 418 K which confirms that this temperature is characterized by a phase transition. The value obtained from the coefficient (*γ*) at the level of the two temperature domains is in the order of 0.0047 K^−1^ for *T* < *T*_tr_ and 0.058 K^−1^ for *T* > *T*_tr_. We noticed that the parameter *γ* depends on the frequency and it increased with an increase in temperature, which makes it possible to identify that the volume of the crystal decreases for *T* > *T*_tr_.^50^

#### 3.6.2 Temperature dependence of the Raman at half maximum

The width of the Raman lines of a photon related to the order–disorder mechanism as a function of the correlation time (*τ*_c_) can be obtained^51^ from the generalized Langevin eqn (14):14
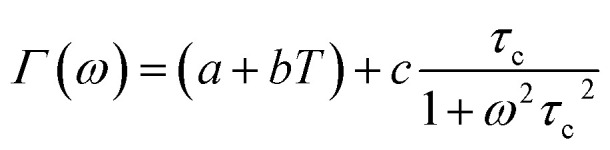
where *τ*_c_ is the correlation time (the time necessary to jump from one potential to another); it is related to orientational activation energy given by eqn (15):15
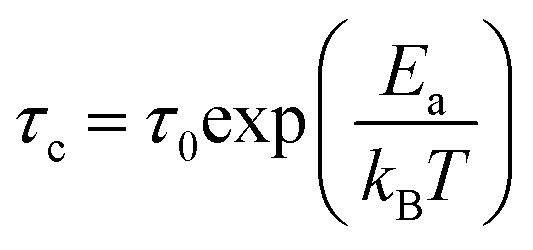
where *τ*_0_ represents the value of *τ*_c_ at high temperature, *E*_a_ is the activation energy for the concerned mode with the order–disorder transition, and *k*_B_ is the Boltzmann constant. Generally, the term (*ωτ*_c_^2^) ≫ 1 so that eqn (14) can be reduced^52^ to eqn (16):16
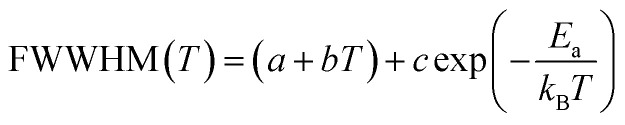
in which *a*, *b*, *c*, and *E*_a_ are parameters that should be fitted.

**Fig. 16:**
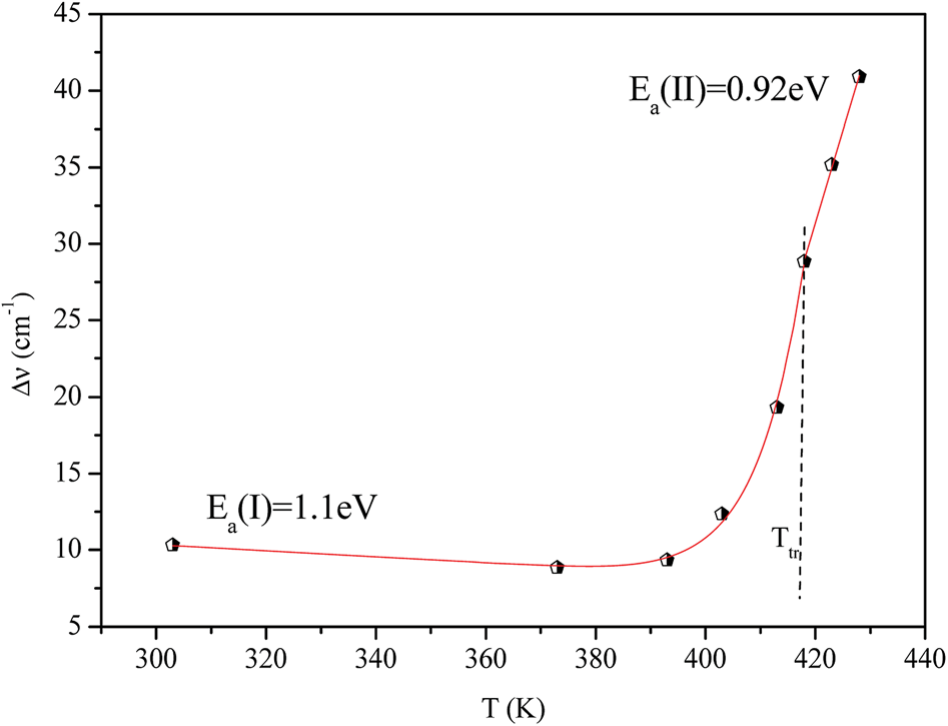
Temperature dependence of the band half-widths at 1440 cm^−1^.

Fig. 16 shows the adjustment of the width at half maximum of the band at 1440 cm^−1^ using eqn (16). This variation is characterized by an enlargement at high temperature due to the reorientation dynamics of the disordered crystal. The activation energies determined from this spectrum are in the order of *E*_a_ (I) = 1.1 eV for *T* < *T*_tr_ and *E*_a_ (II) = 0.92 eV for *T* > *T*_tr_ which are in good agreement with the activation energies obtained by electrical conductivity.

The decrease of these values with increasing temperature is explained by the gradual evolution from an ordered state to a disordered phase resulting from an increase of the dynamics [(C_2_H_5_)_4_N]^+^ cation.^53^ This allows us to conclude that the observed phase transition is related to the reorientation dynamical disorder of the tetraethylammonium cation.

## 4. Conclusion

Tetraethylammonium tetrachloroferrate with a hexagonal system (*P*6_3_*mc* space group) was prepared and investigated as a function of temperature and frequency. One phase transition at 413 K was detected by differential scanning calorimetry (DSC). Additionally, Nyquist plots revealed the existence of grains and grain boundaries that were fitted by an equivalent circuit. The AC conductivity spectrum was found to obey Jonscher's power law.

The temperature dependence of the exponents *s*(*T*) indicated that a correlated barrier hopping (CBH) was the most suitable model for the conduction mechanism. Significant changes of several bands for the internal modes of the organic cation were observed within the phase transition temperature. Temperature dependence analysis of the frequency and the width of the *δ*_as_(CH_2_) mode proved that there is an anomaly around *T* = 418 K, a consequence of an order–disorder phase transition. The decrease of *E*_a_ values below the order–disorder phase transition can be understood as a change in the dynamical states of the cation.

## Conflicts of interest

There are no conflicts to declare.

## Supplementary Material
